# Assessment of chronic liver disease using shear wave elastography: Correlation with serum markers

**DOI:** 10.6026/973206300214772

**Published:** 2025-12-15

**Authors:** Soumik Pal, Rajeev Kumar Ranjan, Suresh Kumar Toppo, Mohd Ismail, Harish Shivprasad Gupta, Sonali Priyadarsini Reddy, Md Shahrukh Ansari

**Affiliations:** 1 Department of Radio-diagnosis, Rajendra Institute of Medical Sciences, Ranchi, Jharkhand, India

**Keywords:** Chronic liver disease, shear wave elastography, liver fibrosis, serum markers, APRI, FIB-4, non-invasive assessment

## Abstract

Chronic liver disease requires accurate fibrosis assessment, with non-invasive tools gaining importance over biopsy. Hence, this cross- sectional study evaluated liver fibrosis in 122 CLD patients using shear wave elastography (SWE) and correlated findings with serum markers. SWE showed strong correlation with APRI (r=0.70) and platelet count (r=-0.70), and moderate correlations with AST and FIB-4. Optimal SWE cutoffs were 9.2 kPa for significant fibrosis and 15.38 kPa for cirrhosis, with excellent diagnostic accuracy (AUROC=0.99). SWE combined with serum markers-especially APRI-provides a reliable, non-invasive alternative for fibrosis assessment in CLD patients.

## Background:

Chronic liver disease (CLD) affects approximately 2 million people globally each year, representing a substantial healthcare burden [1]. The progression of CLD involves continuous inflammation, hepatocyte damage, and regeneration, ultimately leading to fibrosis and cirrhosis [2]. Liver fibrosis, characterized by excessive deposition of extracellular matrix components, is a critical determinant of clinical outcomes, treatment decisions, and prognosis in CLD patients [3]. The traditional gold standard for assessing liver fibrosis has been liver biopsy; however, this invasive procedure carries risks of complications including bleeding, pain, and sampling errors due to the heterogeneous distribution of fibrosis within the liver [4]. These limitations have prompted the development and validation of non-invasive methods for fibrosis assessment, including serum biomarkers and imaging-based techniques [5]. Shear wave elastography (SWE) has emerged as a promising ultrasound-based technique that quantifies tissue stiffness by measuring the propagation speed of shear waves through the liver parenchyma [6]. This technology provides real-time, quantitative assessment of liver stiffness, which correlates strongly with the degree of fibrosis [7]. Several studies have demonstrated the diagnostic accuracy of SWE in staging liver fibrosis across various etiologies of CLD, including viral hepatitis, alcoholic liver disease, and non-alcoholic fatty liver disease [8, 9]. Serum markers, including routine liver function tests (LFTs) and derived indices such as the aspartate aminotransferase-to-platelet ratio index (APRI) and fibrosis-4 (FIB-4) score, offer additional non-invasive approaches for fibrosis assessment [10]. These markers are readily available, cost-effective, and have been validated in multiple populations [11]. However, their diagnostic performance varies across different etiologies and stages of liver disease [12]. Recent studies have explored the combined use of elastography and serum markers to improve diagnostic accuracy for liver fibrosis [13, 14]. Li Y *et al.* reported correlations between Child-Pugh and MELD scores with serum markers in cirrhotic patients, suggesting a link to liver dysfunction [15]. Similarly, Krawczyk *et al.* established correlations between serum markers, histological findings, and liver stiffness measurements [16]. Therefore, it is of interest to quantify liver fibrosis with SWE in patients with CLD. Secondary objectives included comparing SWE measurements with LFTs to explore the relationship between structural changes and functional impairment, and staging liver fibrosis using both SWE and serum markers.

## Materials and Methods:

## Study design and setting:

A cross-sectional study was conducted over 18 months at the Department of Radiodiagnosis, Rajendra Institute of Medical Sciences (RIMS), Ranchi, India. The study protocol was approved by the Institutional Ethics Committee (Memo no. 108 IEC, RIMS dated 27.01.2024), and written informed consent was obtained from all participants.

## Study population:

Patients admitted to the Medicine Department of RIMS, Ranchi, with CLD detected through medical history, clinical examination, or biochemical tests were included in the study. These patients were subsequently referred to the Department of Radiodiagnosis for ultrasound examination.

## Inclusion and Exclusion Criteria:

Inclusion criteria were: (1) patients diagnosed with CLD; (2) age range 18-65 years; (3) adequate liver function test results available for analysis.

Exclusion criteria were: (1) acute liver disease or acute exacerbation of chronic liver disease; (2) pregnancy or breastfeeding; (3) co-existing liver malignancy; (4) known or suspected liver abscess or active liver infection; (5) severe ascites that may interfere with accurate measurement of liver stiffness; (6) inability to undergo SWE examination due to technical limitations.

## Sample size:

Using consecutive sampling, a sample size of 122 participants was determined based on prevalence data from a multicenter study on chronic liver disease in India.

## Shear wave elastography procedure:

SWE measurements were performed using a Phillips Affiniti 70 ultrasound system with a convex transducer (C5-1). The ElastPQ elastography method was employed for stiffness measurements. Patients were positioned either supine or in a left lateral decubitus position with the right arm abducted. Standard gray-scale sonography was initially performed to assess liver echotexture. For SWE, an intercostal approach was used with the patient holding their breath. A region of interest (ROI) free of vessels and ducts was identified on the gray-scale image. The ROI, pre-set to 0.5 cm by 1.5 cm, was positioned at least 1.5-2 cm away from the liver capsule. Stiffness measurements were recorded in kilopascals (kPa) as Young's modulus. Ten consecutive SWE measurements were obtained for each patient, and the device calculated and displayed the average stiffness value. Based on SWE measurements, patients were classified according to METAVIR fibrosis stages using established threshold values: F0-F1 (<8.6 kPa), F2 (8.6-10.46 kPa), F3 (10.46-14.00 kPa), and F4 (>14.00 kPa).

## Serum marker analysis:

Blood samples were collected from all participants for analysis of liver function tests, including total bilirubin, alanine transaminase (ALT), aspartate transaminase (AST), alkaline phosphatase (ALP), gamma-glutamyl transferase (GGT), platelet count, and prothrombin time (PT).

Additionally, the APRI and FIB-4 indices were calculated using the following formulas:

APRI = (AST ÷ AST upper limit of normal) ÷ platelet x 100, where the upper limit was considered to be 33 U/L.

FIB-4 = (age x AST) ÷ (platelet count x √ALT), where age in years, AST and ALT in units per liter, and platelet count in 10^9^ per liter.

Based on APRI and FIB-4 scores, patients were categorized into three groups: no significant fibrosis (F0-F1), significant to advanced fibrosis (F2-F3), and cirrhosis (F4). APRI cutoff values of 0.5 and 1.5, and FIB-4 cutoff values of 1.45 and 3.25 were used for this classification.

## Statistical analysis:

Statistical analysis was performed using SPSS version 26.0 and Microsoft Excel. Categorical variables were presented as frequencies and percentages. Continuous variables were expressed as mean ± standard deviation. Pearson correlation coefficients were calculated to assess the relationship between SWE values and serum markers. Bland-Altman plots were generated to evaluate agreement between SWE and serum markers. Receiver operating characteristic (ROC) curves were constructed to determine the optimal cutoff values of SWE for differentiating fibrosis stages, with APRI and FIB-4 serving as reference standards. A p-value <0.05 was considered statistically significant.

## Results:

A total of 122 patients with CLD were included in the study. The mean age was 44.61±12.08 years, and the mean body mass index (BMI) was 25.25±4.49 kg/m^2^. The study population comprised 72 males (59.02%) and 50 females (40.98%). The etiologies of CLD included alcohol intake in 70 patients (57.38%), non-alcoholic fatty liver disease (NAFLD) in 32 patients (26.23%), and viral hepatitis in 20 patients (16.39%). The mean SWE value was 11.75±3.22 kPa. The descriptive statistics for serum markers and fibrosis indices are presented in Table 1 (see PDF). The mean values were as follows: ALT 31.62±9.29 U/L, AST 43.52±13.74 U/L, PT 12.9±1.35 seconds, bilirubin 0.93±0.29 mg/dL, platelet count 211.05±69.58x10^9^/L, ALP 98.37±26.01 U/L, GGT 61.25±23.81 U/L, APRI 0.76±0.56, and FIB-4 2.05±1.59. The distribution of patients according to METAVIR fibrosis stages based on SWE measurements is shown in Table 2 (see PDF). Twenty-nine patients (23.77%) were classified as F0-F1 (mean SWE 7.89±0.53 kPa), 19 patients (15.57%) as F2 (mean SWE 9.20±0.51 kPa), 37 patients (30.33%) as F3 (mean SWE 12.36±1.04 kPa), and 37 patients (30.33%) as F4 (mean SWE 15.46±1.84 kPa). Pearson correlation coefficients between SWE values and serum markers are presented in Table 3 (see PDF). The strongest correlation was observed between SWE and APRI (r=0.70, p<0.001), followed by platelet count (r=-0.70, p<0.001), AST (r=0.61, p<0.001), and FIB-4 (r=0.59, p<0.001). Moderate positive correlations were found between SWE and PT (r=0.39, p<0.001), bilirubin (r=0.37, p<0.001), ALP (r=0.40, p<0.001), and GGT (r=0.39, p<0.001). A weak positive correlation was observed between SWE and ALT (r=0.34, p<0.001).

ROC curves were generated to determine the optimal cutoff values of SWE for differentiating fibrosis stages. When using APRI as the reference standard, the optimal SWE cutoff for differentiating significant fibrosis (F≥2) from no significant fibrosis (F0-F1) was 9.2 kPa, with a sensitivity of 95%, specificity of 100%, positive predictive value (PPV) of 100%, and accuracy of 96% (AUROC=0.99). For differentiating cirrhosis (F4) from significant to advanced fibrosis (F2-F3), the optimal SWE cutoff was 15.38 kPa, with a sensitivity of 80%, specificity of 99%, PPV of 89%, and accuracy of 96% (AUROC=0.93). When using FIB-4 as the reference standard, the optimal SWE cutoff for differentiating significant fibrosis (F≥2) from no significant fibrosis (F0-F1) was 9.91 kPa, with a sensitivity of 85%, specificity of 65%, PPV of 70%, and accuracy of 75% (AUROC=0.79). For differentiating cirrhosis (F4) from significant to advanced fibrosis (F2-F3), the optimal SWE cutoff was 14.87 kPa, with a sensitivity of 64%, specificity of 83%, PPV of 50%, and accuracy of 85% (AUROC=0.79).

## Discussion:

This study evaluated the role of SWE in assessing liver fibrosis in patients with CLD and correlated these findings with serum markers. The results demonstrated strong correlations between SWE measurements and established serum markers, particularly APRI, AST, FIB-4, and platelet count. Additionally, ROC analysis identified optimal SWE cutoff values for differentiating fibrosis stages, with excellent diagnostic accuracy when using APRI as the reference standard. The strong correlation between SWE and APRI (r=0.70, p<0.001) observed in our study is consistent with previous research. Jeong *et al.* reported a significant correlation between SWE and fibrosis severity, demonstrating the utility of SWE for predicting significant and advanced fibrosis, comparable to serum markers [17]. Similarly, Fraquelli *et al.* found that liver stiffness measured by point shear wave elastography (pSWE) was primarily influenced by liver fibrosis, with performance comparable to transient elastography [18]. The moderate to strong correlations between SWE and AST (r=0.61), FIB-4 (r=0.59), and platelet count (r=-0.70) in our study align with findings from other investigations. Cebula *et al.* reported strong positive correlations between pSWE and most biochemical markers, particularly the King's Score [19]. Additionally, Zhou *et al.* demonstrated the high diagnostic value of 2D-SWE for steatohepatitis and fibrosis, outperforming traditional fibrotic scores [20]. The inverse relationship between SWE and platelet count observed in our study (r=-0.70, p<0.001) is particularly noteworthy. This finding is consistent with the pathophysiology of liver fibrosis, where progressive fibrosis leads to portal hypertension and splenic sequestration of platelets, resulting in thrombocytopenia [21, 22]. Our ROC analysis identified optimal SWE cutoff values of 9.2 kPa for significant fibrosis (F≥2) and 15.38 kPa for cirrhosis (F=4) when using APRI as the reference standard. These values are comparable to those reported in previous studies. Jeong *et al.* established threshold values of 8.6 kPa for significant fibrosis (F≥2), 10.46 kPa for advanced fibrosis (F≥3), and 14.00 kPa for cirrhosis (F=4) [17]. Deffieux *et al.* reported similar cutoffs, with 8.9 kPa for significant fibrosis and 10.20 kPa for cirrhosis [23]. The slight variations in cutoff values across studies may be attributed to differences in patient populations, etiologies of liver disease, and reference standards used. The excellent diagnostic accuracy of SWE observed in our study (AUROC=0.99 for differentiating significant fibrosis when using APRI as reference) supports its utility as a non-invasive tool for fibrosis assessment. This finding is consistent with multiple previous studies that have demonstrated the high diagnostic performance of SWE in staging liver fibrosis [24, 25]. Ayonrinde *et al.* compared 2D-SWE with serum-based fibrosis indices and found that 2D-SWE was significantly more accurate [26]. The integration of SWE with serum markers offers a comprehensive approach to CLD assessment, combining structural and functional information. While SWE provides direct assessment of liver stiffness, serum markers reflect various aspects of liver function and injury. This combined approach may enhance diagnostic accuracy and reduce reliance on invasive biopsy procedures, as suggested by several recent studies [27, 28]. Our study has several limitations. The cross-sectional design allowed for only a snapshot assessment, without longitudinal follow-up to evaluate disease progression. The relatively small sample size may limit the generalizability of our findings. Additionally, the absence of liver biopsy data as a reference standard precluded direct comparison with the histopathological gold standard. Furthermore, the predominance of alcohol-related CLD in our study population may limit the applicability of our findings to patients with other etiologies of liver disease. Future research should focus on large, multi-center studies to validate these findings in diverse populations. Longitudinal studies tracking the progression of CLD using SWE at various stages, including pre- and post-treatment would provide valuable insights into the utility of SWE for monitoring disease progression and treatment response. Additionally, comparing SWE with other imaging techniques such as transient elastography, acoustic radiation force impulse imaging, and magnetic resonance elastography would help determine the most cost-effective and accurate approach for fibrosis assessment.

## Conclusion:

Shear wave elastography is a reliable, non-invasive modality for assessing liver fibrosis in chronic liver disease, demonstrating strong concordance with established serum biomarkers. The combination of SWE with biochemical indices enhances diagnostic accuracy and may substantially reduce reliance on invasive liver biopsy. Overall, SWE represents a practical and effective tool for routine clinical evaluation and longitudinal management of patients with chronic liver disease.
